# Desire for Shorter Life Expectancy From a Mental Health Perspective

**DOI:** 10.2188/jea.JE20220197

**Published:** 2023-09-05

**Authors:** Daisuke Nishi

**Affiliations:** 1 Department of Mental Health, Graduate School of Medicine, The University of Tokyo, Tokyo, Japan; 2 Department of Public Mental Health Research, National Institute of Mental Health, National Center of Neurology and Psychiatry, Tokyo, Japan

In this issue, Yokokawa et al report a large population-based prospective cohort study that followed middle-aged Japanese for approximately 25 years and found that shorter desired life expectancy was significantly associated with increased risk of all-cause mortality.^1^ Specifically, there was increased risk of all-cause mortality in the “shorter-than-average life expectancy” group (hazard ratio [HR] 1.12; 95% confidence interval [CI], 1.04–1.21) and, by cause of death, a particularly large risk ratio for death by suicide (HR 2.15; 95% CI, 1.37–3.38). As much as 30.4% of the association between desired life expectancy and all-cause mortality was mediated by unhealthy lifestyle habits, such as smoking, obesity, and physical inactivity.

Previous findings in mental health epidemiology may be helpful in explaining the association between shorter desired life expectancy and suicide. In this study, participants indicated their desired life expectancy by choosing one of three options: longer than, as long as, or shorter than the average life expectancy. The “shorter than” group accounted for 12.2% of the study population (4,875/39,902). However, this study did not examine suicidal ideation at baseline in 1990. A study of 24,819 community residents conducted in 2020 found that 12.0% of the population reported having suicidal ideation.^2^ Although the proportion of people with suicidal ideation might have increased in 2020 due to the COVID-19 pandemic, given the percentages, it is possible that not a small number of study participants who indicated a desire for “shorter-than-average life expectancy” had suicidal ideation, which could partially explain the association between shorter desired life expectancy and future suicide.

Also, the authors note as a limitation that they did not collect information about self-rated health or negative affectivity, such as depression at baseline.^1^ According to a World Mental Health Japan Survey conducted between 2002 and 2006, the 12-month prevalence of common mental disorders was 7.6% and the lifetime prevalence was 20.3% in Japan.^3^ Thus, it is possible that a certain number of participants who indicated a shorter desired life expectancy had a mental disorder at or prior to the time of the survey.

A part of the feeling of not wanting to live longer might be explained by symptoms of posttraumatic stress disorder (PTSD). The Diagnostic and Statistical Manual of Mental Disorders, Fifth Edition published by the American Psychiatric Association lists “a sense of a foreshortened future (eg, does not expect to have a normal life span)” as one of the diagnostic criteria of PTSD.^4^ Although the lifetime prevalence of PTSD is not high,^3^^,^^5^^,^^6^ about 60% of Japanese people have experienced traumatic events in the narrow sense defined by the DSM,^7^ and about 32% have had adverse childhood experiences (ACEs), such as child abuse.^8^ It is believed that more people have subthreshold PTSD symptoms due to the residual long-term effects of past traumatic experiences compared with those who meet the diagnostic criteria for PTSD. It is conceivable that these individuals might desire a shorter life expectancy. Moreover, the possible mechanism for the negative health effects of ACEs, in which unhealthy behaviors lead to the development of physical and mental illness and premature death,^9^ is consistent with this study’s finding that the association between shorter desired life and death is mediated by unhealthy lifestyle.

It is not always easy to screen for suicidal ideation and traumatic experiences, given the possibility that some participants may find it invasive, and even when the screening results are positive, there may be no experts available to provide treatment. A question about desired longevity, such as the one used by Yokokawa et al, may be relatively easier to ask compared with questions about suicidal ideation, so it may be useful in some situations. 

If shorter desired longevity is reflective of suicidal ideation, psychiatric symptoms, and traumatic experiences to some degree, it is important to consider these factors in terms of countermeasures. There are, of course, an extremely large number of factors associated with suicidal ideation; for example, the interpersonal theory of suicide considers thwarted belongingness and perceived burdensomeness to be key contributors to suicidal ideation.^10^ Thwarted belongingness can be understood as a concept similar to loneliness and social isolation. Perceived burdensomeness is the sense that one’s existence is a burden to family, friends, and society.^10^ It is, therefore, desirable to create a society in which people have a sense of belonging and do not feel that they are a burden on others. Health Japan 21 (second term) includes the goal of improving the social environment, for example, by strengthening community ties, and it is hoped that such efforts will continue to expand. It is also important that each person has opportunities to contribute to their local community and to society as a whole.

We also need to examine other social determinants of mental health, as well as social connections. For instance, social disadvantages are strongly associated with poor mental health.^11^ At the individual level, efforts should focus on issues related to maternal and child health, including ACEs, education, poverty, violence, employment and quality of work, and healthy aging.^12^ The dissemination of trauma-informed care is also essential, given the large number of people who have experienced trauma and the need to prevent re-victimization in the context of support.^13^^,^^14^ At the population level, considerations should include economic and commercial disparities, conflicts, cultural and societal differences, and the physical and natural environments.^12^ It is desirable that such efforts progress in cooperation with other health-related goals and other non-health disciplines. All considered, I hope this paper will be a good opportunity for us all to reaffirm the maxim: “no health without mental health.”^15^
